# Next-Generation Target Discovery in ESKAPE Pathogens: An AI-Driven Framework from Omics-Based to Systems-Level Modeling and Clinical Translation

**DOI:** 10.3390/antibiotics15050469

**Published:** 2026-05-06

**Authors:** Eleonora Chines, Adriana Antonina Tempesta, Ludovica Boscarelli, Matteo Francesco Parisi, Lorenzo Marcoccia, Antonino Capillo, Maria Lina Mezzatesta, Caterina Ledda, Marco Chessari, Viviana Cafiso

**Affiliations:** 1 Department of Biomedical and Biotechnological Sciences, University of Catania, 95123 Catania, Italy; eleonora.chines01@universitadipavia.it (E.C.); adriana.tempesta@phd.unict.it (A.A.T.); ludovica.boscarelli@studium.unict.it (L.B.); m.parisi@studium.unict.it (M.F.P.); mezzate@unict.it (M.L.M.); 2 PhD National Program in One Health Approaches to Infectious Diseases and Life Science Research, Department of Public Health, Experimental, and Forensic Medicine, University of Pavia, 27100 Pavia, Italy; 3 Unit of AI and Computer Systems, Department of Engineering, Università Campus Bio-Medico di Roma, 00128 Rome, Italy; l.marcoccia@teleconsys.it; 4 HorAlzon Lab., Teleconsys SpA, 00144 Rome, Italy; a.capillo@teleconsys.it (A.C.); m.chessari@teleconsys.it (M.C.); 5 Occupational Medicine, Department of Clinical and Experimental Medicine, University of Catania, 95123 Catania, Italy; caterina.ledda@unict.it

**Keywords:** artificial intelligence (AI), antimicrobial resistance, ESKAPE pathogens, machine learning, deep learning, target prioritization, next-generation ESKAPE target discovery

## Abstract

**Background:** Antimicrobial resistance (AMR) among ESKAPE pathogens—*Enterococcus faecium*, *Staphylococcus aureus*, *Klebsiella pneumoniae*, *Acinetobacter baumannii*, *Pseudomonas aeruginosa*, and *Enterobacter* spp.—represents a major global health threat and accounts for a substantial proportion of healthcare-associated infections. Their genomic plasticity and adaptive regulatory responses facilitate the rapid emergence and dissemination of resistance and virulence determinants. Artificial intelligence (AI) has emerged as a powerful approach for analyzing large-scale biological datasets and identifying molecular signatures associated with antimicrobial resistance and pathogenicity. **Objectives:** This review examines AI-driven frameworks for predictive target discovery in ESKAPE pathogens, focusing on approaches that leverage genomic and transcriptomic data and extend toward the integration of additional omics layers within network-based and systems-level modeling frameworks. We discuss how AI methods are evolving beyond phenotypic prediction toward more biologically interpretable inference for prioritizing resistance mechanisms, virulence determinants, and candidate antimicrobial targets. **Conclusions and Future Directions:** Current AI applications exploit genomic, transcriptomic, and network-level data to prioritize resistance and virulence determinants and to support antimicrobial discovery, including small molecules and antimicrobial peptides. However, integrative multi-layer modeling and comprehensive experimental validation remain limited. Future advances will depend on improved integration of complementary biological data, enhanced model interpretability, and robust translational validation frameworks to enable clinically actionable AI-guided novel pathogen-targeted next-generation diagnostics, therapeutic and stewardship strategies against ESKAPE pathogens.

## 1. Introduction

The silent pandemic of antimicrobial resistance (AMR) is exerting increasing pressure on global healthcare systems, potentially undermining decades of progress in infectious disease management. As the transition toward a post-antibiotic era accelerates, the prevalence of multidrug-resistant (MDR) infections poses a critical challenge to standard therapeutic protocols. In high-acuity settings, such as intensive care units, the escalating failure rates of frontline empirical therapies are significantly compromising patient outcomes and increasing mortality risks [1].

The ESKAPE pathogens—*Enterococcus faecium*, *Staphylococcus aureus*, *Klebsiella pneumoniae*, *Acinetobacter baumannii*, *Pseudomonas aeruginosa*, and *Enterobacter* spp.—stand at the center of this global crisis [2]. This notorious group of opportunistic bacteria is responsible for a substantial proportion of healthcare-associated infections and has been prioritized by the World Health Organization in the Bacterial Priority Pathogens Lists, with four ESKAPE members classified as ‘critical’ and the remaining two as ‘high-priority’ threats [3,4]. These pathogens can evade nearly all conventional antibiotics, and their success stems not only from their extensive genetic adaptability but also from their ability to remodel regulatory and metabolic circuits in response to therapeutic pressure. Despite extensive genomic surveillance and high-throughput screening efforts, the development of novel antimicrobials continues to lag alarmingly.

In response to this escalating challenge, Artificial Intelligence (AI) has emerged as a key enabling technology, offering a paradigm shift by enabling data-driven discovery of predictive molecular targets that underpin both identification, antimicrobial resistance and pathogenicity [5,6]. Leveraging the vast volumes of biological data generated by genomics, transcriptomics, and proteomics, AI-driven approaches are now enabling the identification of subtle molecular patterns underlying resistance and virulence. Machine learning (ML), a core AI subset, employs statistical algorithms to uncover intricate patterns and relationships within data, enabling robust predictions on new observations. Deep learning (DL) techniques leverage hierarchical neural network architectures to automatically learn feature representations directly from raw data [6].

ML models, exemplified by random forests (RF), support vector machines (SVM), and gradient boosting frameworks such as XGBoost, excel at feature selection from high-dimensional phenotypic and genotypic datasets, identifying key resistance determinants in ESKAPE pathogens [7,8,9].

DL models such as DeepARG [10], DeepAMR [11], and TGC-ARG [12] further advance this capability by predicting antimicrobial susceptibility directly from raw genomic and metagenomic data, identifying both known and novel resistance determinants with superior sensitivity compared to traditional bioinformatics approaches.

Advanced frameworks, including graph neural networks and transformers, extend these capabilities by simultaneously profiling virulence factors and resistance markers through multi-task learning and self-attention mechanisms [13].

In parallel, revolutionary advances in protein structure prediction—exemplified by AlphaFold2 [14], RoseTTAFold [15], and ESMFold [16]—have expanded the frontiers of in silico drug discovery, characterizing protein targets in ESKAPE pathogens. Graph neural networks and knowledge graph embedding models are similarly advancing rational drug design and repurposing by linking genomic data with pharmacological potential. Together, these approaches herald a paradigm shift from descriptive microbiology to predictive, AI-driven discovery.

Although several recent reviews have examined AI-assisted antibiotic discovery or antimicrobial resistance prediction more broadly, fewer have specifically focused on predictive target discovery in ESKAPE pathogens while integrating biological interpretability, validation, and translational readiness into a unified framework.

This review evaluates AI frameworks for predictive target discovery in ESKAPE pathogens, focusing on representative studies integrating genomic and transcriptomic data within multi-omics and network-based approaches. It examines methodological advances that move beyond phenotype prediction toward biologically interpretable identification of resistance mechanisms, virulence determinants, and candidate antimicrobial targets. We also assess the current state of validation, clinical integration, and implementation readiness, providing a perspective on the translational maturity of AI-driven antimicrobial resistance models.

## 2. Methodological Approach

### 2.1. Search Strategy and Selection Criteria

A literature search was conducted to identify studies applying artificial intelligence (AI) approaches to the predictive discovery of antibiotic resistance and virulence determinants in ESKAPE pathogens. Articles were retrieved from PubMed/MEDLINE using combinations of the following keywords: (“artificial intelligence” OR “machine learning” OR “deep learning” OR “neural network” OR “graph neural network” OR “generative model” OR “ensemble learning” OR “QSAR”) AND (“antimicrobial resistance” OR “virulence” OR “target discovery” OR “target prioritization” OR “antimicrobial peptide” OR “drug discovery”) AND (“*Enterococcus faecium*” OR “*Staphylococcus aureus*” OR “*Klebsiella pneumoniae*” OR “*Acinetobacter baumannii*” OR “*Pseudomonas aeruginosa*” OR “*Enterobacter*”). The search was limited to publications from the last decade (2016–2026). A total of 277 records were identified.

**Figure 1:**
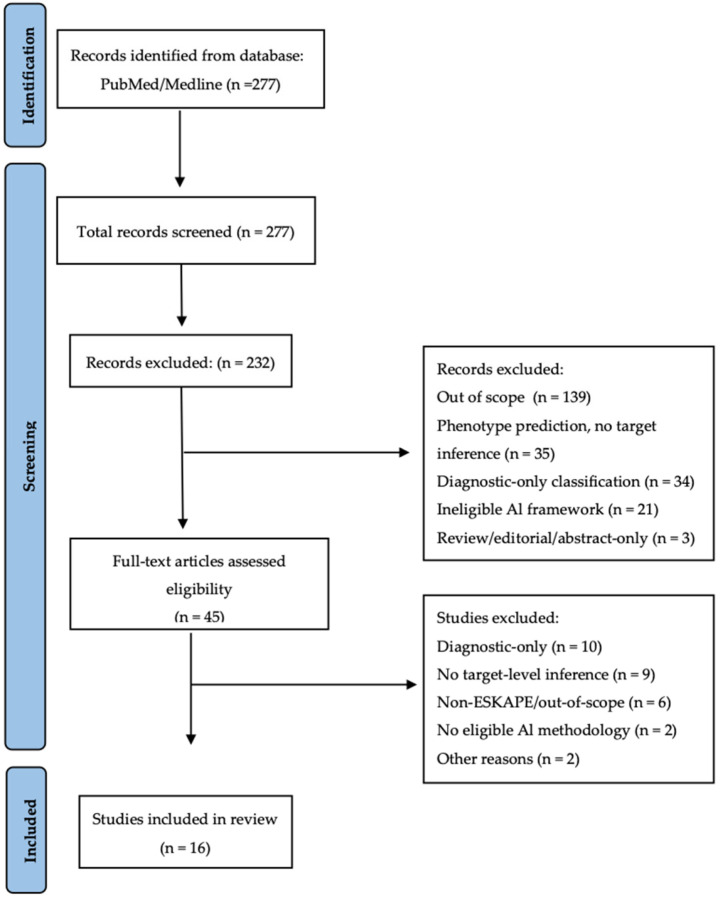
PRISMA-style flow diagram of study selection for AI-based resistance and virulence determinant discovery and antibiotic/antimicrobial peptide discovery in ESKAPE pathogens.

This focused narrative review applied predefined inclusion criteria prioritizing studies that provided functional target inference and/or experimental validation alongside predictive performance, while enabling critical evaluation of their methodological limitations. Sixteen studies were selected as the core analytical corpus, with additional references included to contextualize broader methodological and translational developments. The study identification, screening, eligibility assessment, and inclusion steps are summarized in a PRISMA-style flow diagram [17] (Figure 1).

### 2.2. Inclusion and Exclusion Criteria

Studies were considered eligible if they applied AI-based methodologies (e.g., machine learning (ML), deep learning (DL), ensemble models, or related computational approaches) to investigate antibiotic resistance or virulence determinants in one or more ESKAPE pathogens. Studies on AI-guided antibiotic or antimicrobial peptide discovery were included only when the computational framework explicitly identified or prioritized specific resistance or virulence-associated molecular targets. Articles were excluded if they focused exclusively on diagnostic classification without mechanistic inference. Studies addressing non-ESKAPE pathogens or not employing AI-based methods were also excluded. Reviews, editorials, and conference abstracts were not considered. Studies limited to phenotypic resistance prediction were included only when biological insight or target-level interpretation was provided. Only publications in English were included.

### 2.3. Study Selection and Data Extraction

All retrieved records were screened at the title and abstract level to determine eligibility. Screening and full-text assessment were performed independently by two reviewers, with disagreements resolved through consensus. Of the 277 screened records, 45 articles were assessed in full text, and 16 studies met all eligibility criteria. For each included study, data were extracted on pathogen species, biological data type (e.g., whole-genome sequencing, transcriptomics, chemical libraries, or peptide libraries), feature representation, AI architecture and associated computational tools, primary predictive objective, level of target inference (e.g., sequence-, gene-, or network-level), validation strategy, and reported limitations. Given the methodological heterogeneity of AI architectures, datasets, and validation strategies across studies, no formal risk-of-bias tool was applied. Instead, studies were qualitatively evaluated with attention to dataset provenance, interpretability strategies, validation approaches, and the presence of experimental confirmation.

## 3. AI-Driven Frameworks for Predictive Target Discovery in ESKAPE Pathogens

**Figure 2:**
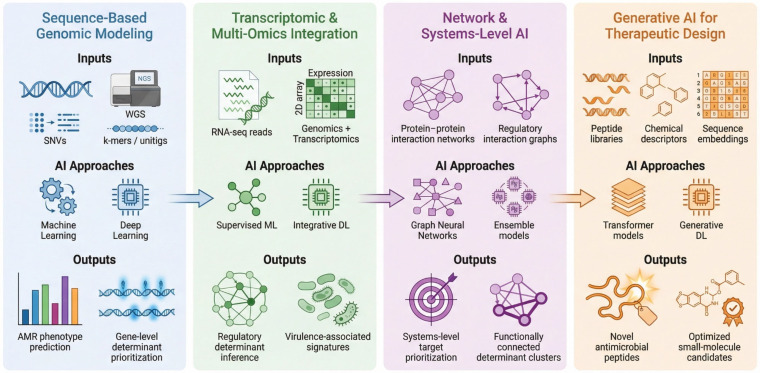
Conceptual multi-layer AI framework for predictive target discovery in ESKAPE pathogens. The framework is organized across four interconnected layers. Sequence-based genomic modeling provides the foundation for resistance prediction and gene-level determinant prioritization using features derived from whole-genome sequencing (e.g., SNVs, k-mers). Transcriptomic and multi-omics integration captures dynamic regulatory processes associated with resistance and virulence. Network and systems-level approaches further integrate these signals to identify functionally connected targets, while generative AI frameworks translate these insights into antimicrobial design, enabling the development of novel peptides and small-molecule candidates. Together, these layers reflect a progression from data-driven prediction toward increasingly mechanistic and translational inference.

Artificial intelligence has enabled multi-layered strategies for predictive target discovery in ESKAPE pathogens, as shown in Figure 2. Current approaches encompass sequence-based genomic modeling, transcriptomic and multi-omics inference, network-level systems modeling, and AI-guided therapeutic compound discovery. Collectively, these frameworks spanned the continuum from resistance determinant prioritization to mechanistic interpretation and translational antimicrobial development.

### 3.1. Sequence-Based AI Models for Resistance Determinant Prioritization

Sequence-based AI models have been extensively applied to genomic datasets to predict antimicrobial resistance (AMR) phenotypes and to prioritize candidate resistance determinants [18,19,20,21]. Across these studies, different genomic feature representations were employed, including gene presence/absence matrices derived from whole-genome sequencing (WGS), single-nucleotide variant (SNV) profiles, and high-resolution k-mer or unitig-based sequence embeddings. The choice of representation critically influences both predictive performance and interpretability depth. Hyun et al. implemented an interpretable ML framework combining support vector machine classifiers with a random subspace ensemble strategy (SVM-RSE), using gene presence/absence matrices extracted from WGS data to associate genomic content with resistance phenotypes in *S. aureus*, *P. aeruginosa*, and *E. coli* [18]. Rather than focusing exclusively on classification performance, the framework leveraged feature selection and ranking to identify genomic signatures linked to antimicrobial resistance, enabling prioritization of candidate genes and genomic regions for downstream investigation. Avershina et al. developed the AMR-Diag framework, which employs feed-forward artificial neural networks trained on k-mer-based pan-genomic features to predict β-lactam resistance (e.g., ampicillin, third-generation cephalosporins, and carbapenems) in *E. coli* and *K. pneumoniae*. Specifically, the authors developed a database of β-lactamase-associated k-mers (BLAKs) extracted from known resistance genes. Feature selection was performed using Neighboring Component Analysis (NCA) prior to model training, enabling the identification of informative genomic determinants associated with resistance phenotypes [19]. This assembly-free encoding allowed rapid capture of genomic sequence variation directly from raw reads, while the NCA-derived feature weights enabled model outputs to be associated with specific β-lactamase gene families (e.g., NDM, CTX-M, TEM). Li et al. applied ML models—including ensemble and boosting algorithms such as Random Forest and extreme gradient boosting (XGBoost)—trained on whole-genome sequencing (WGS) data represented as k-mer features to predict imipenem resistance in *K. pneumoniae*. The framework enabled genome-wide detection of resistance-associated sequence signatures, demonstrating that high-resolution k-mer representations can capture both known and previously uncharacterized genetic determinants linked to carbapenem resistance [20]. Jia et al. extended sequence-based modeling by integrating WGS-derived resistance determinants with gene expression measurements in *A. baumannii* [21]. Genomic features, including antimicrobial resistance genes and mobile genetic elements identified using tools such as ResFinder and ISsaga, were used as inputs for the model. Deep neural network models were trained to estimate minimum inhibitory concentrations (MICs), and the incorporation of transcriptional data enabled the identification of resistance-associated gene signatures beyond genomic variation alone.

Many of the identified genomic determinants are known to be associated with mobile genetic elements, including plasmids, transposons, and integrons, which play a central role in the horizontal transfer and dissemination of antimicrobial resistance across ESKAPE pathogens.

While these approaches achieve high predictive performance, several limitations remain. Sequence-based models are often sensitive to dataset composition, including class imbalance and lineage-specific biases, which may affect generalizability. In addition, k-mer-based representations, although powerful for capturing sequence variation, can limit biological interpretability and mechanistic attribution compared to gene-level features, making it more challenging to directly link predictions to functional resistance mechanisms. Furthermore, high model accuracy does not necessarily imply causal inference, highlighting the need for systematic validation and integration with complementary data modalities.

### 3.2. Transcriptomic AI Frameworks for Resistance and Virulence Regulatory Inference

While sequence-based genomic AI models rely predominantly on static genetic variation, transcriptomic frameworks enable interrogation of dynamic regulatory states underlying antimicrobial resistance and virulence. Kula et al. developed transcriptome-based ML models using supervised classifiers such as logistic regression, Random Forest, XGBoost, and Naive Bayes to predict antimicrobial resistance phenotypes in *P. aeruginosa*, while also identifying transcriptomic signatures associated with resistance [22]. RNA sequencing profiles were used as predictive features, and differential expression analysis integrated with model-derived feature importance enabled the identification of transcriptional signatures associated with resistance phenotypes, thereby anchoring inference in regulatory activity rather than gene presence alone.

Transcriptomic AI approaches have also been applied to virulence determinant prioritization. Yu et al. applied RNA sequencing and independent component analysis (ICA) to infer virulence-associated transcriptional modules in *S. aureus*, identifying the small RNA Sau-41 as a regulatory element linked to pathogenicity [23]. Gene weights derived from the ICA components enabled prioritization of candidate regulatory determinants involved in virulence regulation. These elements were subsequently validated through molecular interaction assays (EMSA), haemolysis experiments, and a murine infection model. This study illustrates how AI-based transcriptomic decomposition can guide experimental validation, linking computational prediction with mechanistic characterization of virulence regulation. Similarly, Artini et al. applied supervised ML classifiers, including support vector machines, Random Forest, decision trees, and gradient boosting models, to chemical composition and biofilm modulation datasets of *P. aeruginosa*, including isolates from cystic fibrosis patients [24,25]. These studies associated specific gene expression patterns with virulence and biofilm phenotypes and confirmed prioritized candidates through experimental validation. Biofilm formation represents a major virulence determinant across ESKAPE pathogens. In phenotype-driven biofilm studies, supervised models were employed to establish quantitative activity–composition relationship frameworks, identifying chemical components associated with biofilm inhibition at sub-bactericidal concentrations [24,25]. Although these models do not directly infer gene-level regulatory mechanisms, they demonstrate how AI can prioritize modulators of virulence-associated phenotypes and generate biologically testable hypotheses regarding pathways involved in sessile adaptation and biofilm regulation.

These transcriptional signatures are often associated with known biological processes, including stress-response pathways, efflux pump regulation, and virulence-associated regulatory networks, which are key contributors to antimicrobial resistance and pathogenicity in ESKAPE pathogens.

Beyond transcriptomic inference, proteomic profiling provides an additional functional layer by directly quantifying protein abundance, post-translational regulation, and the activity of resistance-associated pathways. In bacterial pathogens, including ESKAPE species, antibiotic exposure can induce rapid remodeling of membrane proteins, efflux systems, and virulence determinants that may not be fully captured at the transcriptional level. Recent studies have highlighted how mass-spectrometry-based proteomics can reveal functional responses to antimicrobial stress and identify protein-level biomarkers associated with resistance and pathogenicity [26,27,28]. Although several studies have explored the integration of ML with mass-spectrometry-based proteomics to predict antimicrobial resistance or support rapid microbial identification, these approaches are primarily oriented toward diagnostic classification rather than mechanistically interpretable target discovery. Consequently, proteomics-based studies were not represented among the core articles included in this review, which specifically focused on AI-driven frameworks for predictive target prioritization in ESKAPE pathogens.

Despite their ability to capture dynamic regulatory states more directly than sequence-based models, transcriptomic AI frameworks present several limitations. Model performance is strongly influenced by experimental variability, including batch effects, growth conditions, and data normalization strategies, which may reduce reproducibility and cross-study generalizability. Moreover, while these approaches identify expression-based signatures associated with resistance or virulence, distinguishing causal regulatory mechanisms from correlated transcriptional patterns remains challenging. The integration of transcriptomic data with complementary multi-omics layers and standardized validation frameworks will be essential to improve robustness, biological interpretability, and reliable target-level inference.

### 3.3. Network-Based and Systems-Level AI Frameworks for Target Prioritization

Network-based and systems-level AI frameworks enable deeper biological interpretation by incorporating explicit functional relationships among genes and proteins. By modeling regulatory and protein–protein interaction networks, these approaches move beyond individual feature ranking and allow inference at the level of coordinated functional modules and interaction clusters. Burrows et al. applied non-negative matrix factorization (NMF) to accessory gene presence–absence matrices to resolve the structural organization of the *Enterobacter* pan-genome and identify gene sets associated with lineage-specific and horizontally transferred traits [29]. By decomposing the accessory genome into distinct gene modules, termed Phylons, the framework enabled the classification of major lineage-associated and mobile genetic elements across the genus. Although primarily focused on pan-genome structure rather than direct phenotype prediction, the study illustrates how unsupervised ML can support biologically informative target and trait prioritization beyond conventional classification tasks. Kim et al. applied supervised ML classifiers, including logistic regression, Random Forest, and gradient-boosted decision trees, to whole-genome sequencing data from *E. faecium* and *E. faecalis* in order to identify genomic determinants associated with antimicrobial resistance. Model-dependent and model-independent feature selection, including Block HSIC Lasso, enabled the prioritization of candidate resistance drivers and highlighted the importance of interpretability in distinguishing biologically meaningful predictors from potential confounding signals [30]. By comparing multiple genomic feature representations, including AMR genes, pangenome features, and predicted plasmid clusters, the study showed that ML can recover both known resistance determinants and additional candidate features potentially linked to resistance-associated mobile genetic elements. Despite strong predictive performance, the authors also demonstrated that careful inspection of top-ranked features is necessary to avoid overinterpreting signals driven by population structure rather than direct resistance mechanisms.

A more explicit systems-level representation is achieved through graph-based architectures. Zhang et al. applied supervised ML models, including logistic regression, Random Forest, XGBoost, support vector machines, and k-nearest neighbors, to transcriptomic datasets in order to identify genes associated with the transition of *S. aureus* from planktonic growth to biofilm formation [31]. Recursive feature elimination was subsequently employed to prioritize a reduced subset of key genes contributing to the classification model. Functional enrichment analyses indicated that several of these genes were linked to metabolic pathways such as urea metabolism and arginine biosynthesis, while downstream biofilm-protein prediction suggested potential roles for previously uncharacterized proteins in biofilm regulation. These findings illustrate how machine learning-based feature selection can refine large transcriptomic datasets to identify candidate determinants associated with complex pathogenic phenotypes. Overall, these approaches extend AI modeling from individual features toward the analysis of functional relationships and interaction networks.

These network-derived modules frequently correspond to known biological processes, including metabolic adaptation, quorum sensing, and biofilm formation, which are key determinants of persistence and antimicrobial tolerance in ESKAPE pathogens.

Network-based and systems-level approaches can improve interpretability but remain dependent on the quality and completeness of underlying interaction data, which are often inferred and highly context-specific. Consequently, identified modules may capture indirect associations rather than true functional relationships. In addition, signals driven by population structure or feature co-occurrence can be misinterpreted as mechanistic links if not carefully controlled. Distinguishing causal interactions from correlated network patterns, therefore, remains a key challenge for reliable target prioritization and mechanistic interpretation.

### 3.4. AI-Guided Antibiotic and Antimicrobial Peptide Discovery

Artificial intelligence is increasingly applied to the discovery and optimization of novel antimicrobial agents, including small molecules and antimicrobial peptides (AMPs). In contrast to determinant-prioritization frameworks, these approaches primarily focus on predicting antibacterial activity at the compound or sequence level, using chemical descriptors, structural information, or peptide sequence embeddings as model inputs. Recent advances in protein structure prediction, including AlphaFold-based models, may further support structure-informed characterization of antimicrobial targets and facilitate structure-guided compound prioritization, complementing data-driven AI discovery pipelines. Landmark studies have demonstrated the potential of deep learning-based screening frameworks to identify structurally novel antibiotics, exemplified by the discovery of halicin through large-scale neural network screening of chemical libraries [32].

Boulaamane et al. implemented a machine learning-guided workflow integrating QSAR modeling with molecular docking to prioritize compounds active against *A. baumannii*, focusing on the outer membrane protein OmpW as a potential therapeutic target. Molecular fingerprints were used to encode chemical structures, and several classifiers—including Random Forest, support vector machines, k-nearest neighbors, Gaussian Naive Bayes, and convolutional neural networks—were evaluated for activity prediction and virtual screening of candidate molecules [33]. In vitro validation confirmed antibacterial activity and supported target engagement through complementary assays, partially bridging phenotypic prediction and mechanistic hypothesis generation. Similarly, AI-guided screening strategies have enabled the identification of pathogen-specific compounds such as abaucin, a narrow-spectrum antibiotic targeting *A. baumannii* identified through machine learning-based virtual screening approaches [34].

Antimicrobial peptide discovery represents an even more active domain of AI-driven antimicrobial development. Sequence-based DL architectures trained on curated AMP datasets have been widely employed to learn sequence–activity relationships and generate optimized peptide candidates. In many cases, these training datasets derive from curated antimicrobial peptide libraries that compile experimentally identified peptides from diverse biological sources, including microbial organisms, plants, and animal host-defense systems [35,36]. Proteomic and peptidomic investigations based on mass spectrometry have contributed substantially to the identification and characterization of naturally occurring antimicrobial peptides and host-defense peptides, thereby expanding the repertoire of sequences available for computational modeling [37]. Such experimentally derived peptide libraries provide valuable resources for training machine learning models aimed at predicting antimicrobial activity and optimizing peptide design. However, despite this potential, most AI-driven antimicrobial peptide discovery studies currently rely primarily on curated sequence databases rather than large-scale proteomic datasets, indicating that proteomics-based peptide discovery remains an underexplored resource for data-driven antimicrobial development. Li et al. and Bolatchiev et al. utilized neural network-based frameworks trained on antimicrobial peptide libraries to learn sequence–activity relationships and guide antimicrobial peptide design [38,39]. These recurrent neural network architectures enabled the generation of novel candidate peptides, which were subsequently validated experimentally against representative ESKAPE pathogens. Notably, Bolatchiev et al. further demonstrated the in vivo efficacy of selected peptides in an experimental sepsis model, highlighting the translational potential of AI-guided antimicrobial peptide discovery. Mishra et al. implemented an ML-guided peptide optimization strategy integrated with experimental validation in murine infection models targeting *S. aureus* [40], thereby strengthening translational relevance. Similarly, Zhao et al. applied a deep generative framework to design pathogen-targeted antimicrobial peptides, exploring previously uncharacterized sequence space while enforcing physicochemical and antimicrobial constraints, thereby expanding candidate diversity beyond conventional AMP libraries [41]. Their approach combines a conditional variational autoencoder with a diffusion-based generative model and an integrated MIC prediction module, enabling the generation and prioritization of peptides with programmable physicochemical properties and predicted activity against specific pathogens. More broadly, such generative strategies rely on architectures such as recurrent neural networks, transformer-based models, or diffusion frameworks to navigate high-dimensional peptide sequence landscapes under learned biological constraints. Complementing these generative approaches, recent integrated ML workflows have successfully identified potent AMPs specifically targeting multidrug-resistant ESKAPE pathogens, such as *A. baumannii* and *S. aureus*, demonstrating high efficacy in both in vitro assays and in vivo skin infection models [42]. These AI-prioritized compounds and peptides are generally consistent with known antimicrobial mechanisms, including membrane disruption, interference with essential cellular processes, and modulation of host–pathogen interactions, although such mechanisms are not always explicitly resolved by the models. However, mechanistic interpretability remains limited across most AMP studies. While AI effectively optimizes physicochemical properties for potency, explicit mapping of the specific Mechanism of Action (MOA)—such as linkage to internal regulatory networks or non-canonical molecular targets—is frequently absent. Bridging generative antimicrobial design with systems-level resistance modeling, therefore, represents a critical next step toward unifying predictive target discovery and AI-driven therapeutic innovation within ESKAPE pathogens.

AI-driven antimicrobial discovery shows strong potential, but several limitations remain. Many frameworks rely predominantly on in silico evaluation, with limited experimental validation beyond small candidate sets. In addition, training datasets—particularly for antimicrobial peptides—are often biased toward known sequences, which may limit generalization and the exploration of novel sequence space. For generative models, linking predicted activity to specific mechanisms of action remains challenging. Bridging these gaps will be essential for translating computational predictions into clinically relevant antimicrobial candidates.

Taken together, these approaches illustrate the progressive evolution of AI frameworks from phenotype prediction toward more mechanistically informed target discovery in ESKAPE pathogens. While current models successfully capture associations between genetic variation, regulatory activity, and functional interactions, most approaches remain predominantly based on genomic or transcriptomic data and rely on supervised learning paradigms. The integration of multi-omics data and robust experimental validation remains limited, highlighting the need for more comprehensive and translationally oriented frameworks. Within this context, a major cross-cutting challenge underlying these frameworks is the biological interpretability of AI predictions—an issue that has motivated the increasing adoption of explainable artificial intelligence approaches.

A structured overview of the core studies analyzed in this review—including pathogens, data modalities, AI architectures, and specific computational tools—is presented in Table 1. This synthesis specifically details the Interpretability Methods (XAI) employed to transition from predictive modeling toward target-level inference and experimental validation strategies. To complement this qualitative overview and address the heterogeneity of reported approaches, we further compiled a descriptive quantitative summary of representative studies (Table 2), including dataset size, model type, and reported performance metrics. The different predictive tasks addressed (e.g., AMR prediction, MIC estimation, or antimicrobial discovery) are explicitly indicated to contextualize the reported metrics, allowing a comparative overview of performance ranges across model classes while acknowledging their limited direct comparability.

### 3.5. Bridging the Interpretability Gap: The Role of eXplainable AI (XAI) in Target Discovery

While the previous Section 3.1, Section 3.2, Section 3.3 and Section 3.4 highlight the predictive performance of AI frameworks applied to antimicrobial resistance and virulence inference, translating these outputs into biologically meaningful insights requires the integration of explainable artificial intelligence (XAI) approaches. In this context, interpretability represents a critical bridge between computational prediction and target-level understanding.

The evolution of interpretability can be observed in the transition from early confidence scoring approaches [38] to more advanced strategies, including knowledge graph-guided optimization [40] and property-preserving generative frameworks [41]. These developments have paved the way for feature attribution methods such as SHAP and LIME [43,44], which enable a quantitative assessment of how multiple features contribute to antimicrobial resistance predictions and associated phenotypic outcomes.

Beyond genomic markers, XAI approaches must increasingly account for dynamic functional layers. As highlighted by Aita et al. [26], genomic data define the genetic basis of resistance but often fail to capture real-time protein-level adaptations. This limitation is further compounded by cellular heterogeneity, where emerging approaches such as single-cell proteomics require interpretability frameworks capable of distinguishing population-level resistance from stochastic tolerance [27]. In this context, metabolic modeling represents an important extension of XAI, moving from static biomarkers to functional fluxomics. Peng et al. [28] demonstrated that AI can identify specific metabolic bottlenecks associated with resistance—such as the suppression of the pyruvate cycle—effectively providing a roadmap for metabolic adjuvant therapy to restore antibiotic susceptibility through targeted reprogramming. Interpretability also plays a central role in mechanism-of-action (MOA) inference. Frameworks such as CoHEC and the Clairvoyance algorithm [45] enable the decomposition of complex predictions into interpretable decision paths, facilitating the identification of novel MOA profiles, including those associated with compounds such as darobactin. The development of standardized evaluation frameworks remains essential to ensure that these predictions can be translated into biologically meaningful and experimentally testable targets [46].

Ultimately, interpretability is emerging as a fundamental design principle in AI-guided antimicrobial discovery, supporting the transition from predictive modeling toward mechanistically informed and clinically relevant target identification.

## 4. Translational and Clinical Implementation Considerations

**Figure 3:**
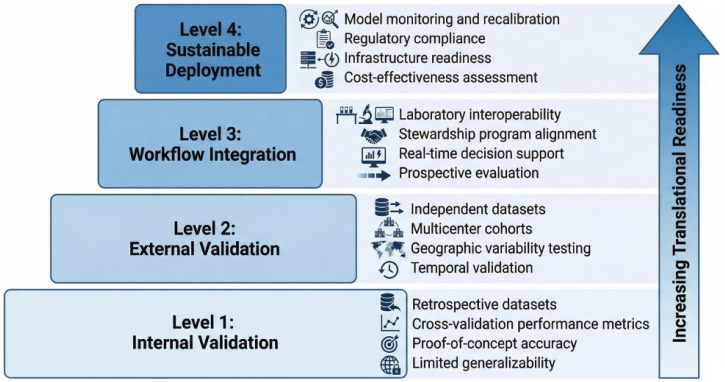
Translational maturity framework for AI-driven antimicrobial resistance models.

Despite rapid advances in AI for AMR prediction and target prioritization, translating these computational advances into routine clinical practice remains challenging. Predictive accuracy alone is insufficient; real-world implementation depends on validation, reproducibility across institutions, workflow integration, interpretability, and infrastructural readiness. As illustrated in Figure 3, progression from retrospective model development to durable clinical deployment demands sequential advancement across validation, integration, governance, and monitoring.

### 4.1. Validation and Generalizability Across Clinical Settings

Many AI-based AMR studies rely on retrospective datasets evaluated through internal cross-validation. While suitable for proof-of-concept development, such approaches do not ensure robustness across heterogeneous healthcare environments [47,48]. Because resistance epidemiology is geographically and temporally dynamic, evaluation beyond internal cross-validation is critical for clinical credibility.

Recent multicenter studies have begun to address this gap. Yong et al. [49] conducted a randomized multicenter investigation integrating MALDI-TOF mass spectrometry with ML for rapid screening of methicillin-resistant *S. aureus*, demonstrating reproducible performance across independent laboratories. Rocchi et al. [50] evaluated ML models for predicting *K. pneumoniae* resistance across multiple Italian centers, highlighting both feasibility and variability in cross-site performance. Similarly, Wang et al. [51] compared ML algorithms for predicting multidrug-resistant organism (MDRO) infections in a multicenter cohort, revealing performance differences linked to institutional case mix. Lee et al. [52] further demonstrated performance variability in a dual-center validation study of multidrug-resistant urinary tract infection prediction. Collectively, these findings indicate that multicenter validation is a necessary but still insufficient step toward translational readiness.

Models trained on single-center datasets risk overfitting to local epidemiology and laboratory practices, potentially limiting transferability across healthcare systems. Moreover, dataset shift and temporal variability can significantly affect model performance over time, underscoring the need for continuous monitoring and recalibration strategies [53].

### 4.2. Integration into Clinical Microbiology and Stewardship Workflows

Robust validation alone does not guarantee clinical adoption. Even models demonstrating robust performance across multicenter validation settings may fail to translate into routine practice if they are not operationally compatible with laboratory turnaround times, diagnostic infrastructure, and antimicrobial stewardship workflows [54,55]. Without structured integration, predictive systems remain confined to research environments despite demonstrated performance.

Prospective implementation studies provide preliminary evidence of feasibility. Elligsen et al. [56] evaluated an ML-guided decision support system for empiric therapy in Gram-negative bacteremia within a prospective clinical framework, demonstrating integration into routine care. Gómez De La Torre et al. [57] reported real-world applications of an ML-based clinical decision support system in bacteremia management, emphasizing interoperability with electronic health records and stewardship oversight.

At the laboratory level, Hu et al. [58] developed an mNGS-based ML model for rapid antimicrobial susceptibility testing of *A. baumannii*, illustrating how validated genomic prediction models can be embedded into diagnostic workflows. Similarly, Azami et al. [59] described a Nanopore-based sequencing workflow (BacT-Seq) for pathogen identification and resistance prediction directly from positive blood cultures, highlighting practical considerations related to sequencing infrastructure, data pipelines, and turnaround time. These approaches build upon ongoing efforts to integrate whole-genome sequencing into clinical microbiology laboratories, including applications in outbreak investigation and routine typing [60].

Translational success, therefore, remains contingent on the ability to embed validated models within interoperable, stewardship-aligned clinical infrastructures. Despite these advances, real-world implementations remain limited and are largely confined to pilot or research settings.

### 4.3. Interpretability, Regulatory Requirements, and Clinical Accountability

Interpretability represents a critical determinant of clinical adoption. In antimicrobial stewardship and resistance prediction, algorithmic outputs must be transparent and clinically contextualized. Black-box models, even when accurate, may face resistance if their predictions cannot be linked to recognizable clinical or microbiological variables [61].

Reporting standards such as CONSORT-AI [62] aim to improve transparency and methodological rigor in clinical AI research. In addition, risk-of-bias appraisal tools for prediction models, such as PROBAST [63], support structured evaluation of methodological quality and applicability.

At the model level, Yang et al. [64] demonstrated how feature attribution methods can enhance interpretability in antibiotic effectiveness prediction. Goldschmidt et al. [65] further highlighted the importance of clinician oversight in ML-based antibiotic appropriateness assessment within intensive care settings.

Regulatory requirements further shape deployment. AI systems guiding antimicrobial therapy are likely to be regulated as software-as-a-medical-device frameworks, requiring reproducibility, validation, and post-deployment monitoring [65,66]. Continuous surveillance mechanisms are therefore essential to detect model drift and preserve patient safety. Ultimately, AI-driven AMR models must augment clinical expertise without displacing professional judgment, while preserving accountability, governance, and patient safety. These requirements further underscore that most AI-driven AMR systems remain at an early stage of clinical translation.

### 4.4. Structural Barriers to Sustainable Deployment

Beyond methodological considerations, structural constraints may limit large-scale implementation. Dataset representativeness remains a central concern, as many models are developed using data from high-income healthcare systems, potentially restricting applicability across diverse epidemiological settings and introducing risks of algorithmic bias [47,67,68]. Temporal variability in resistance patterns introduces additional challenges related to dataset shift and model drift, necessitating ongoing recalibration [50]. Infrastructural and economic limitations—including sequencing capacity, computational resources, and integration with laboratory information systems—may further constrain deployment [58,59]. Finally, the absence of harmonized benchmarking frameworks, standardized reporting, and formal bias assessment tools continues to limit reproducibility and cross-study comparability [48,63,69]. Addressing these systemic barriers will be essential to prevent fragmentation of AI initiatives and to ensure equitable, durable implementation across heterogeneous healthcare systems.

## 5. Limitations and Future Perspectives

Future developments in AI-driven antimicrobial resistance research will likely emphasize multi-modal data integration, scalable validation frameworks, and improved generalizability across healthcare systems. Despite these advances, most currently available models still rely on single-layer datasets—predominantly genomic or transcriptomic data—while comprehensive multi-omics integration remains relatively rare. In addition, many models are affected by dataset biases, including class imbalance, population structure, and limited geographic diversity, which may constrain generalizability across clinical settings. The incorporation of genomic, transcriptomic, and clinical metadata may enhance mechanistic insight and predictive robustness beyond single-layer models [70]. Another critical challenge concerns model interpretability and the distinction between predictive associations and causal mechanisms, which remains a major limitation for clinical translation. Federated learning approaches could support multicenter model development while addressing data privacy and representativeness challenges [71]. Future work should also prioritize standardized benchmarking frameworks and harmonized evaluation metrics to improve reproducibility and cross-study comparability. Ultimately, sustained clinical impact will depend on prospective evaluation, adaptive model updating strategies to account for evolving resistance patterns, and alignment with global antimicrobial resistance surveillance initiatives [52,66].

## 6. Conclusions

AI is reshaping antimicrobial resistance research in ESKAPE pathogens, enabling predictive prioritization of resistance and virulence determinants and accelerating antimicrobial discovery. However, methodological sophistication alone does not ensure clinical impact. Sustainable translation requires rigorous validation, interpretability, and accountable implementation within healthcare systems [72]. In light of the substantial and rising global burden of bacterial antimicrobial resistance projected through mid-century [73], the ultimate value of AI will depend on its capacity to bridge predictive modeling with mechanistic insight and real-world stewardship integration.

## Figures and Tables

**Table 1:** Summary of studies evaluating AI-driven frameworks for predictive target discovery in ESKAPE pathogens.

Ref.	First Author (Year)	Pathogens	Data Type	Feature Representation	AI Architecture	Tools	Interpretability Method (XAI)	Primary Objective	Target-Level Inference	Experimental Validation
[18]	Hyun (2020)	*S. aureus* *P. aeruginosa* *E. coli*	WGS	Gene presence/ absence matrix	Supervised ML	SVM + RSE	RFE + Feature Weighting	AMR prediction	Gene-level	Internal cross-validation
[19]	Avershina (2021)	*K. pneumoniae* *E. coli*	WGS	K-mers (BLAKs database)	DL	Feed-forward ANN	NCA	AMR prediction	Sequence-level	Internal cross-validation
[20]	Li, S. (2023)	*K. pneumoniae*	WGS	k-mer genomic features	Supervised ML	RF; LR; SVM; GBDT; XGBoost	Chi-squared + Gini Importance	AMR prediction	Genome-wide feature-level	External validation (independent dataset)
[21]	Jia (2024)	*A. baumannii*	WGS + Transcriptomics	Resistance genes + expression levels	DL	DNN integrating WGS and transcriptomic features	SHAP + Cohen’s Kappa	MIC prediction	Gene-level (integrative)	In vitro validation (MIC assays)
[22]	Kula (2026)	*P. aeruginosa*	Transcriptomics (RNA-seq)	Gene expression matrix	Supervised ML	RF; LR; XGBoost; NB	ANOVA + RF Importance + DESeq2	AMR prediction	Gene-level (regulatory)	Internal cross-validation
[23]	Yu (2023)	*S. aureus*	Transcriptomics	RNA-seq signatures	Unsupervised ML	ICA	iModulon Decomposition	Virulence prioritization	Gene-level	In vitro mechanistic and in vivo validation
[24]	Artini (2022)	*P. aeruginosa*	Chemical composition + biofilm phenotypic data	Essential oil composition profiles	ML classification	RF; LR; DT; GB; SVM;; *k*NN	FI + PDP	Virulence/Biofilm	Phenotype-level	In vitro phenotypic validation
[25]	Artini (2018)	*P. aeruginosa*	Chemical + biofilm phenotypic data	Essential oil composition profiles	ML classification	GB	FI + PDP	Virulence/Biofilm	Phenotype-level	In vitro phenotypic validation
[29]	Burrows (2026)	*Enterobacter* spp.	Genomic data	Gene presence/ absence matrix	Unsupervised ML	NMF	Phylons (Gene clusters)	Pangenome structure/AMR	Gene-level	Computational validation
[30]	Kim (2024)	*E. faecium* *E. faecalis*	Genomic data	Gene/ Plasmid presence-absence	Supervised ML	LR; RF; GBDT	MDI + Block HSIC Lasso	AMR prediction	Operon/ Transposon level	Internal cross-validation
[31]	Zhang (2024)	*S. aureus*	Transcriptomics	Gene expression signatures	Supervised ML	RF; LR; XGBoost; SVM; *k*NN	RFE + Network Modules	Virulence prioritization	Gene-level	Internal cross-validation
[33]	Boulaamane (2024)	*A. baumannii*	Chemical screening	Molecular descriptors + docking	ML	RF; SVM; *k*NN; GNB; CNN	SAR + Docking	Drug discovery	Target-level (OmpW)	In vitro antibacterial assays
[38]	Li, C. (2024)	*E. coli* *S. aureus*	Peptide library	Sequence descriptors	DL	RNN	CS + Similarity Analysis	AMP discovery	Sequence-level	In vitro antibacterial assays
[39]	Bolatchiev (2022)	*K. pneumoniae* *P. aeruginosa*	Peptide library	Physicochemical features	DL	RNN	MD + AlphaFold	AMP discovery	Sequence-level	In vitro and in vivo validation
[40]	Mishra (2025)	*S. aureus*	Peptide library	Physicochemical descriptors + peptide sequence patterns	ML optimization	k-means clustering; knowledge graph-guided sequence optimization	AGO Patterns + t-SNE	AMP discovery	Sequence-level	In vivo validation (murine model)
[41]	Zhao (2025)	*E. coli* *S. aureus*	Peptide library	Generative sequence modeling	Generative DL	CVAE + conditional diffusion model	Property Preservation Loss	AMP design	Sequence-level	In silico validation

**Legend:** Whole-Genome Sequencing **(WGS)**; RNA sequencing **(RNA-seq)**; Machine Learning **(ML)**; Deep Learning **(DL)**; Antimicrobial Resistance **(AMR)**; Antimicrobial Peptide **(AMP)**. Support Vector Machine **(SVM)**; Random Subspace Ensemble **(RSE)**; Artificial Neural Network **(ANN)**; Neighboring Component Analysis **(NCA)**; Random Forest **(RF)**; Logistic Regression **(LR)**; Extreme Gradient Boosting **(XGBoost)**; Gradient Boosting Decision Tree **(GBDT)**; Deep Neural Network **(DNN)**; Naive Bayes **(NB)**; Independent Component Analysis **(ICA)**; Decision Tree **(DT)**; Gradient Boosting **(GB)**; *k* Nearest Neighbors **(*k*NN)**; Non-negative Matrix Factorization **(NMF)**; Gaussian Naive Bayes **(GNB)**; Convolutional Neural Network **(CNN)**; Recurrent neural network **(RNN)**; Conditional Variational Autoencoder **(CVAE)**; Recursive Feature Elimination **(RFE);** Differential Gene Expression **(DGE);** Feature Importance **(FI);** Partial Dependence Plots **(PDP)**; Mean Decrease in Impurity **(MDI)**; Structure-Activity Relationship **(SAR)**; Confidence Scoring **(CS)**; Molecular Dynamics **(MD)**; Amino Acid Group Occurrence **(AGO)**; eXplainable AI **(XAI)**.

**Table 2:** Synthesis of dataset size, model type, and reported performance across AI studies.

Ref.	Study	Task	Dataset Size	Model Type	Evaluation Metrics	Reported Performance
[18]	Hyun et al., 2020	AMR prediction	288 *S. aureus*; 456 *P. aeruginosa*; 1588 *E. coli* genomes	SVM-RSE	Accuracy, AUC, MCC	Acc: 79.3–99.5%, AUC: 0.79–1.00, MCC: 0.39–0.95
[19]	Avershina et al., 2021	AMR prediction	90 *E. coli*; 76 *K. pneumoniae* isolates	FFNN	Accuracy	Acc: 94–100% (WT/NWT), 91–99% (S/R)
[20]	Li et al., 2023	AMR prediction	1 WGS data *K. pneumoniae*	RF, LR, SVM, GBDT, XGBoost	AUC	AUC: 0.965–0.969
[21]	Jia et al., 2024	MIC prediction	518 clinical *A. baumannii* isolates + 1978 public genomes	DNN + LR	Accuracy, MIC within ±1 dilution	DNN Acc: 87.6–98.6%, MIC Acc: 86.2% (±1 dilution)
[22]	Kula et al., 2026	AMR prediction	414 *P. aeruginosa* isolates	RF, LR, XGBoost, NB	Accuracy, Sensitivity, Specificity	Acc: 77.6–98.8%, Sens: 68.4–100%, Spec: 78.8–95.8%
[23]	Yu et al., 2023	Virulence inference	506 *S. aureus* RNA-seq datasets	ICA	N/A	Regulatory module identified (Sau-41)validated experimentally
[24]	Artini et al., 2022	Virulence/Biofilm prediction	8 *P. aeruginosa* strains	RF, LR, DT, GB, SVM, kNN	Accuracy, MCC, F1-score	Acc: 0.69–0.98, MCC: 0.35–0.88, F1: 0.59–0.99
[25]	Artini et al., 2018	Virulence/Biofilm prediction	1 *P. aeruginosa*	GB	MCC	MCC: ~0.40–0.60
[29]	Burrows et al., 2026	Genomic structure inference	777 *Enterobacter* genomes	NMF	Accuracy, FPR, FPR	Acc: 90.5%, FPR: 0.01, FPR: 0.30
[30]	Kim et al., 2024	AMR prediction	309 *E. faecium*, 338 *E. faecalis*	LR, RF, GBDT	Accuracy	Acc: 89.0–99.1%
[31]	Zhang et al., 2024	Virulence prediction	175 *S. aureus* RNA-seq samples	RF, LR, XGBoost, SVM, kNN	Accuracy, AUC	Acc: 83–94%, AUC: 0.92–0.97
[33]	Boulaamane et al., 2024	Drug discovery	3196 compounds, 11,648 screened	RF, SVM, kNN, GNB, CNN	AUC, Accuracy, MCC	AUC: 0.84–0.96, CNN AUC: 0.96, Acc: ~0.90; MCC: ~0.80
[38]	Li et al., 2024	AMP generation	2253 training, 20,000 generated, 58 tested	RNN	Accuracy	Acc: 95.5–100%
[39]	Bolatchiev et al., 2022	AMP generation	3100 training, 198 generated, 5 tested	RNN	Hit rate, MIC	Hit rate: 40%, MIC: 2–8 μg/mL, in vivo survival up to 66.7%
[40]	Mishra et al., 2025	AMP optimization	14,743 AMPs	k-means + knowledge graph	MIC, biofilm reduction	MIC: 4–8 μg/mL, biofilm ↓ 3–4 log_10_; in vivo MRSA ↓ 2.3 log_10_
[41]	Zhao et al., 2025	AMP generation	2.3M UniProt fragments; 4546 AMPs (GRAMPA)	CVAE + diffusion	MSE, MAE, MIC	MSE: 0.343–0.381, MAE: 0.440–0.463, MIC < 20 µM: 65.1% (*E. coli*), 85.0% (*S. aureus*)

**Legend:** Antimicrobial Resistance **(AMR)**; Minimum Inhibitory Concentration **(MIC)**; Area Under the Curve **(AUC)**; Matthews correlation coefficient **(MCC)**; False Positive Rate (FPR); False Negative Rate **(FNR)**; Random Forest **(FR)**; Logistic Regression **(LR)**; Support Vector Machine **(SVM)**; Gradient Boosting Decision Tree **(GBDT)**; Extreme Gradient Boosting **(XGBoost)**; Naive Bayes **(NB)**; Gaussian Naive Bayes **(GNB)**; k-nearest neighbors **(kNN)**; Decision Tree **(DT)**; Gradient Boosting **(GB)**; Convolutional Neural Network **(CNN)**; Recurrent Neural Network **(RNN)**; Feed-Forward Neural Network **(FFNN)**; Deep Neural Network **(DNN)**; Independent Component Analysis **(ICA)**; Non-negative Matrix Factorization (NMF); Conditional Variational Autoencoder **(CVAE)**. Giant Repository of AMP Activities **(GRAMPA)**; Universal Protein Resource **(UniProt)**, Not applicable **(N/A)**. ↓ indicates a decrease.

## Data Availability

No new data were created or analyzed in this study.
